# Biological Augmentation of Arthroscopic Partial‐Thickness Bursal‐Sided Rotator Cuff Repair Using Minced Autologous Glenohumeral Ligament Tissue Particles

**DOI:** 10.1002/atn2.70348

**Published:** 2026-11-03

**Authors:** Hengzhi Liu, Xinji Wang, Huajie Mao, Weiliang Shen

**Affiliations:** ^1^ Department of Sports Medicine & Orthopedic Surgery, The Second Affiliated Hospital Zhejiang University School of Medicine Hangzhou Zhejiang P. R. China; ^2^ Institute of Sports Medicine Zhejiang University Hangzhou Zhejiang P. R. China; ^3^ Orthopedics Research Institute of Zhejiang University Hangzhou Zhejiang P. R. China; ^4^ Key Laboratory of Motor System Disease Research and Precision Therapy of Zhejiang Province Hangzhou Zhejiang P. R. China; ^5^ Clinical Research Center of Motor System Disease of Zhejiang Province Hangzhou Zhejiang P. R. China

## Abstract

Rotator cuff tears are associated with high retear rates and limited healing capacity after repair. We describe an arthroscopic biological augmentation technique using minced autologous glenohumeral ligament tissue during double‐row partial‐thickness bursal‐sided rotator cuff repair. Ligament tissue is harvested, processed into particulate grafts, and implanted within a suture‐bridge construct to create a stable, biologically active interpositional layer at the repair site. This technique provides a simple and fully autologous method to enhance the local healing environment during rotator cuff repair.

## Graphical abstract

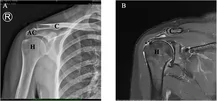

**VIDEO 1:** The patient is placed in the beach‐chair position, and the affected shoulder is evaluated through standard posterior and anterior arthroscopic portals. The video shows a tendon‐preserving technique for a symptomatic bursal‐sided partial‐thickness rotator cuff tear. After diagnostic glenohumeral arthroscopy confirms preservation of the articular‐sided tendon layer and adequate glenohumeral ligament tissue, a limited amount of autologous middle glenohumeral ligament/inferior glenohumeral ligament tissue is selectively harvested without compromising anterior capsuloligamentous stability. The harvested ligament is minced on the back table into 1 to 2 mm^3^ particles, creating a compact autologous particulate graft. In the subacromial space, the bursal‐sided tear and decorticated footprint are prepared, sutures are passed through the intact tendon adjacent to the defect, and the ligament particles are delivered into the superficial tear gap before final suture tightening. Final knot fixation compresses the tendon against the bone bed and contains the graft within a closed suture‐bridge pocket, creating a mechanically protected and biologically active interpositional layer at the repair interface. Video content can be viewed at https://doi.org/10.1002/atn2.70348.

Rotator cuff tears remain a common and challenging musculoskeletal disorder, with high postoperative retear rates and suboptimal healing quality continuing to limit clinical outcomes.^1^ Although small full‐thickness tears often show favorable mechanical stability and biological potential after double‐row repair, bursal‐sided partial‐thickness tears present distinct biological challenges.^2^, ^3^ These lesions are frequently associated with tendon degeneration, chronic subacromial bursitis, reduced vascularity, and diminished collagen organization.^4^ In addition, preservation of residual tendon attachment constrains suture placement and limits tendon‐bone contact, further compromising the intrinsic healing environment.^5^

To address these limitations, we describe an arthroscopic biological augmentation technique using minced autologous glenohumeral ligament tissue. The glenohumeral ligament shares structural and extracellular matrix characteristics with the rotator cuff tendon and can be safely harvested without compromising shoulder stability. Minced ligament particles are implanted within a suture‐bridge construct, creating a stable and biologically active interpositional layer that may enhance the local regenerative microenvironment during rotator cuff repair.

## SURGICAL TECHNIQUE

A video presenting this technique is provided (Video 1).

### Preoperative Planning

All patients undergo clinical evaluation and magnetic resonance imaging of the affected shoulder. Magnetic resonance imaging confirms a symptomatic bursal‐sided partial‐thickness rotator cuff tear refractory to nonoperative treatment and is used to assess tear morphology, tendon quality, and glenohumeral ligament integrity. Patients with imaging evidence of significant anterior shoulder instability are excluded to avoid compromising capsular function (Figure 1).

**FIGURE 1:**
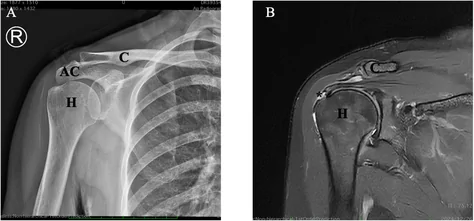
Preoperative imaging evaluation. (A) Anteroposterior radiograph of the affected shoulder obtained with the patient upright, showing the key osseous landmarks used for preoperative orientation, including the AC, C, and H. (B) Coronal oblique fat‐suppressed T2‐weighted MRI of the same shoulder, oriented from lateral to medial across the supraspinatus footprint. The image shows a symptomatic bursal‐sided partial‐thickness tear of the supraspinatus tendon (*), with preservation of the deeper articular‐sided tendon layer over the H. The clavicle (C) is labeled as a superior landmark. Together, the radiograph and MRI establish the diagnosis, side, and anatomic orientation before tendon‐preserving repair with biological augmentation. (AC, acromion; C, clavicle; H, humeral head; MRI, magnetic resonance imaging.)

### Patient Positioning and Diagnostic Arthroscopy

All procedures are performed with the patient under general anesthesia in the beach‐chair position, with the torso elevated approximately 60° to 80°. A standard posterior portal is established, and diagnostic arthroscopy of the glenohumeral joint is performed (Figure 2A). A systematic intra‐articular assessment includes evaluation of the humeral head and glenoid cartilage surfaces, the anterior, posterior, and superior labrum, and the long head of the biceps tendon. Particular attention is directed to the integrity of the superior glenohumeral ligament, middle glenohumeral ligament (MGHL), and inferior glenohumeral ligament (IGHL) (Figure 2B). Once a bursal‐sided partial‐thickness rotator cuff tear (Ellman grade II or III) is confirmed with preserved articular‐sided tendon attachment and satisfactory residual tendon quality, a tendon‐preserving repair strategy is selected (Figure 2C).

**FIGURE 2:**
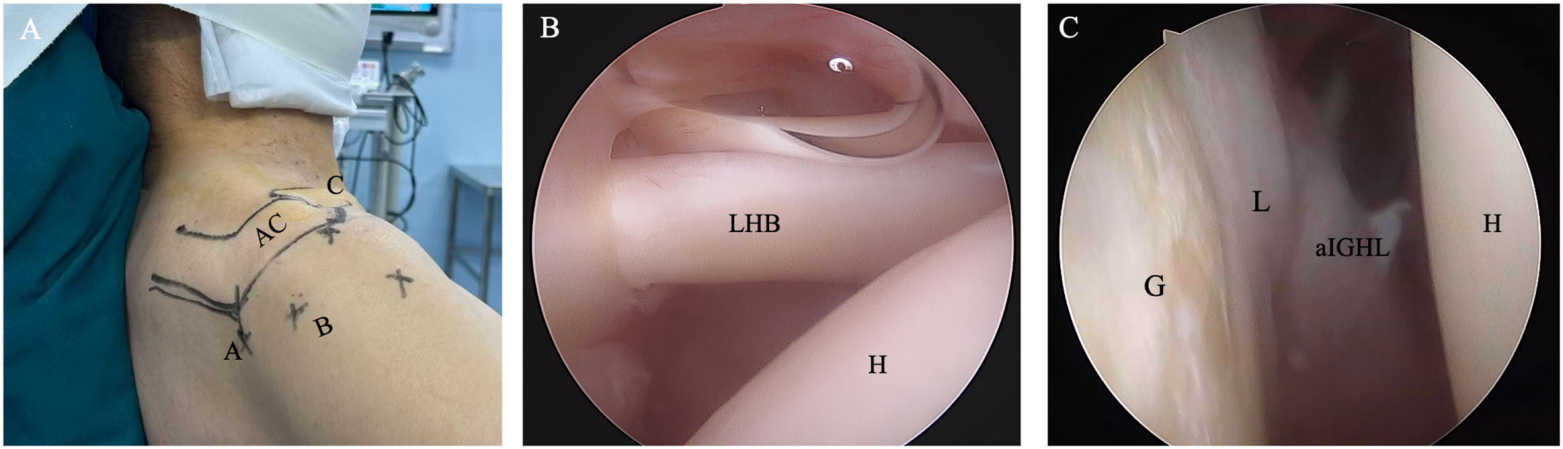
Patient positioning, portal placement, and diagnostic arthroscopy. (A) The affected shoulder is prepared with the patient in the beach‐chair position. Surface landmarks are marked to orient the surgeon to the AC and C, and the planned arthroscopic portals are outlined, including the posterior viewing portal (A) and accessory working portal (B). This setup provides access to both the glenohumeral joint and subacromial space while maintaining a consistent orientation for subsequent tendon‐preserving repair. (B) Diagnostic glenohumeral arthroscopy is performed through the posterior viewing portal. The LHB tendon and H are visualized, providing intra‐articular orientation and confirming the relation between the superior glenohumeral structures and the rotator cuff footprint. (C) With the arthroscope in the posterior viewing portal, the anterior capsuloligamentous complex is inspected. The G, L, aIGHL, and H are labeled. This view emphasizes the need to assess capsuloligamentous integrity before harvesting autologous glenohumeral ligament tissue, because the tissue should be obtained only when adequate ligament quality is present and shoulder stability will not be compromised. (AC, acromion; aIGHL, anterior band of the inferior glenohumeral ligament; C, clavicle; G, glenoid; H, humeral head; L, labrum; LHB, long head of the biceps.)

### Evaluation and Preparation of Minced Ligament Tissue

Through the anterior portal, the MGHL and IGHL are probed to assess fiber orientation and tension. Ligament tissue is considered suitable for harvest when the MGHL or IGHL shows normal morphology or mild hypertrophy without compromising anterior shoulder stability; transection of the taut anterior band of the IGHL is carefully avoided (Figure 3A). Approximately 1 to 2 cm^2^ of ligament tissue is harvested using an arthroscopic punch or motorized shaver (Arthrex, Naples, FL) (Figure 3B). In cases of reduced tissue elasticity, multiple small cuts followed by grasping of ligament fragments may facilitate tissue retrieval (Figure 3C).

**FIGURE 3:**
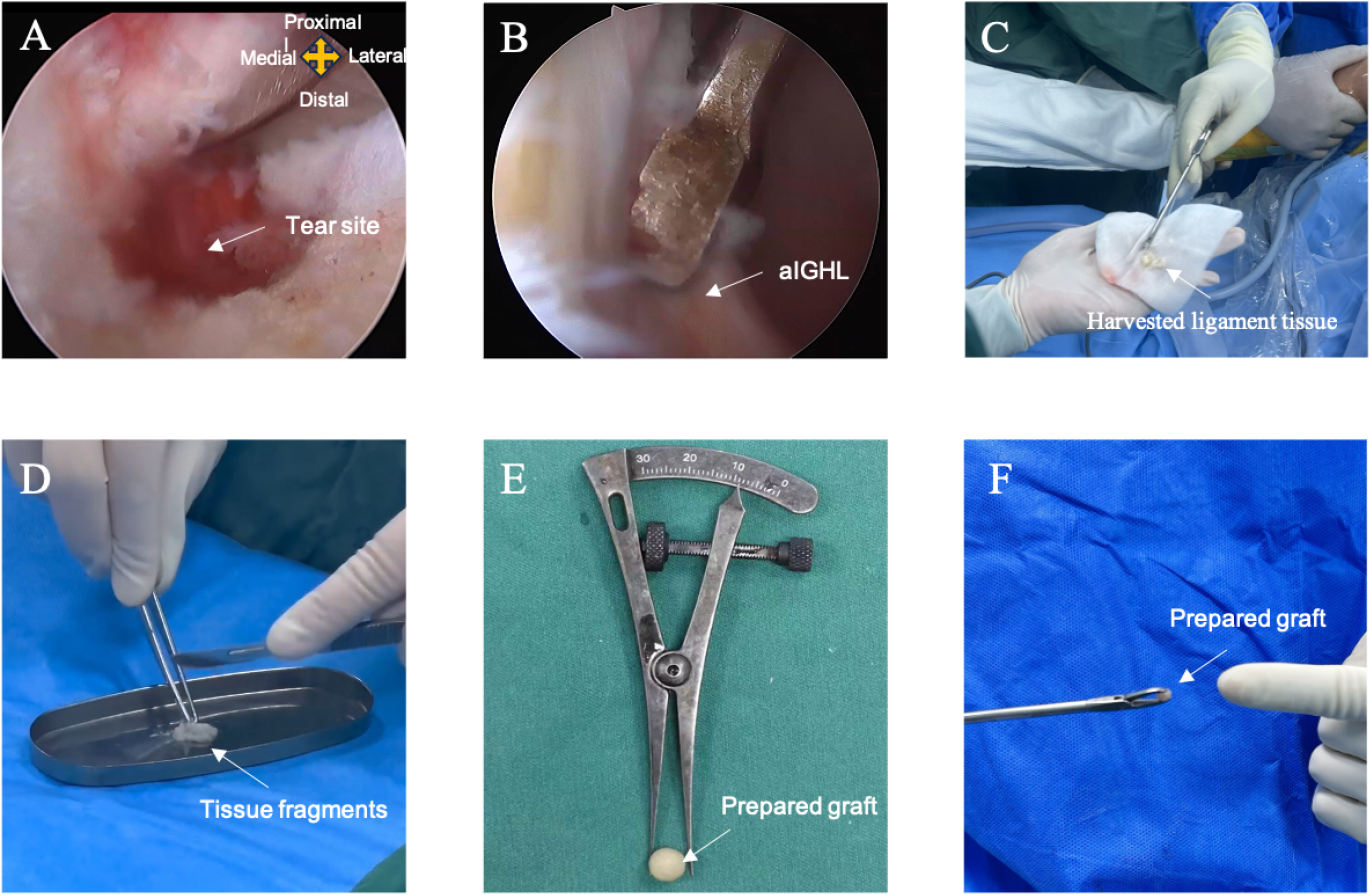
Harvesting and preparation of minced autologous glenohumeral ligament particles. (A) Arthroscopic intra‐articular view of the affected shoulder with the patient in the beach‐chair position, obtained through the posterior viewing portal. The orientation marker indicates proximal, distal, medial, and lateral directions. The bursal‐sided partial‐thickness rotator cuff tear is confirmed without full‐thickness perforation, and the tear site is identified before proceeding with biological augmentation. (B) Through the anterior working portal, a limited portion of suitable glenohumeral ligament tissue is harvested under direct arthroscopic visualization. The aIGHL is labeled. (C) The harvested ligament tissue is retrieved from the joint and transferred to the sterile operative field. (D) On the sterile back table, the harvested tissue is blotted and minced into small fragments using a scalpel while the tissue is stabilized with forceps. The goal is to convert the ligament into small tissue fragments suitable for arthroscopic delivery. (E) The tissue fragments naturally coalesce into a compact prepared graft after mincing. (F) The prepared autologous ligament particle graft is loaded onto an arthroscopic grasper and reserved for implantation. Glenohumeral ligament tissue can be processed into an autologous particulate scaffold that is small enough for arthroscopic delivery but cohesive enough to be contained within the repair construct. (aIGHL, anterior band of the inferior glenohumeral ligament.)

The harvested tissue is transferred to a sterile stainless‐steel tray on the back table, where excess fluid is gently blotted to prevent slippage. While stabilizing one end of the tissue with sterile forceps, the specimen is minced using a scalpel in a crisscross fashion; microsurgical scissors may be used to release residual fibers (Figure 3D). The tissue is processed into 1 to 2 mm^3^ fragments, which naturally coalesce into a particulate graft measuring approximately 5 to 9 mm in diameter and is reserved for implantation (Figure 3E and F).

### Modified Double‐Row Repair With Ligament Particle Implantation

The bursal surface of the torn tendon is gently debrided using a shaver to remove degenerative tissue and loose fibers while preserving healthy tendon. The corresponding footprint is lightly decorticated with a shaver or curette to create a bleeding bone bed without violating the intact articular‐sided tendon attachment.

Based on tear size, a medial anchor is placed near the footprint margin, and sutures are passed through the intact tendon adjacent to the bursal‐sided tear to achieve horizontal compression (DePuy Mitek, Raynham, MA). For wider tears, an additional lateral anchor is inserted to create a modified double‐row configuration (Figure 4A). Before final tensioning of the sutures, the preprepared minced autologous glenohumeral ligament particles are delivered through the anterior working portal using a grasper and placed into the superficial tear gap between the tendon and the bone bed (Figure 4B). Because the deep tendon layer is preserved, the particulate graft is distributed primarily along the decorticated footprint beneath the bursal‐sided defect. The particles are inserted incrementally to ensure even distribution and to avoid graft aggregation or dead space formation. After graft placement, the sutures are gradually tensioned using a sliding knot technique to compress the tendon against the bone bed while stabilizing the interposed ligament particles. Final fixation is achieved with nonsliding knots, creating a structurally stable, tendon‐preserving repair with a biologically active interpositional interface. Tendon tension and graft stability are reassessed arthroscopically to confirm the absence of graft extrusion or suture cut‐through (Figure 4C). Subacromial decompression or acromioplasty is performed when indicated. The procedure concludes with thorough irrigation and standard portal closure. Surgeons may refer to Table 1 for helpful clinical pearls and pitfalls.

**FIGURE 4:**
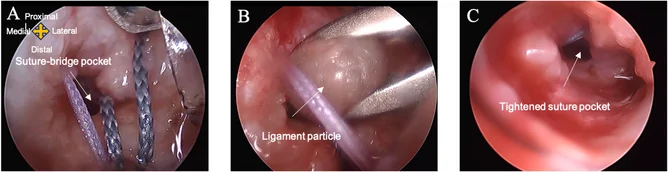
Particulate graft implantation and tendon‐preserving modified double‐row repair. (A) Sutures are passed through the intact tendon adjacent to the bursal‐sided partial‐thickness tear, and a modified double‐row construct is created with a medial anchor (and an additional lateral anchor for wider tears) to provide horizontal compression. (B) The preprepared minced autologous glenohumeral ligament particles are delivered through the anterior working portal using a grasper and placed into the superficial tear gap between the tendon and the decorticated footprint before final suture tensioning. (C) After gradual tensioning and final knot fixation, arthroscopic reassessment confirms stable tendon compression and graft containment without extrusion or suture cut‐through.

**TABLE 1:** Pearls and Pitfalls for Arthroscopic Rotator Cuff Repair Augmented With Autologous Glenohumeral Ligament Tissue Particulates

**Pearls**	**Pitfalls**
Use of autologous glenohumeral ligament tissue as a biological augment avoids immunologic rejection and provides excellent biocompatibility	Considerable interpatient variability in glenohumeral ligament development, thickness, and tension may limit tissue availability in some cases because of anatomic variation
Ligament tissue minced into 1 ‐ 2 mm^3^ particles naturally aggregates into 5 ‐ 9 mm clusters, preserving a 3‐dimensional scaffold and retaining endogenous growth factors (e.g., TGF‐β and VEGF) that may support angiogenesis and collagen deposition	Ligament particle implantation must be completed before final suture tensioning; the limited working space and narrow time window require advanced arthroscopic skill and efficient team coordination
Implantation of ligament particles within a closed interpositional layer created by a modified double‐row suture construct allows combined mechanical compression and biological scaffold stabilization, reducing the risk of graft migration and dead space formation	Uneven particle distribution or formation of graft aggregation and dead space may adversely affect the quality of the healing interface; incremental delivery with continuous arthroscopic visualization is essential
Harvesting ligament tissue from regions with preserved anatomy and moderate tension ( MGHL or IGHL), while avoiding critical stabilizing structures, enables tissue reuse without compromising shoulder stability	

IGHL, inferior glenohumeral ligament; MGHL, middle glenohumeral ligament; TGF‐β, transforming growth factor beta; VEGF, vascular endothelial growth factor.

### Postoperative Rehabilitation

A standardized home‐based rehabilitation program is initiated immediately after surgery, including pendulum, elbow, and wrist exercises. From 2 to 6 weeks, patients are limited to passive shoulder range of motion and grip exercises while wearing a sling except during approved activities. Assisted active and active shoulder range of motion, along with scapular exercises, are gradually introduced between 6 and 12 weeks, with resistance and isometric strengthening beginning at approximately 10 weeks. From 3 to 12 months postoperatively, patients progress toward full range of motion and advanced strengthening.

### Ethical Approval

The study was approved by the Ethics Review Committee of the Second Affiliated Hospital of Zhejiang University, and all patients obtained informed consent.

## DISCUSSION

Bursal‐sided partial‐thickness rotator cuff tears present unique biological and technical challenges despite preservation of residual tendon attachment.^6^ Compared with small full‐thickness tears, these lesions are frequently associated with tendon degeneration, chronic subacromial inflammation, and limited vascularity, which may compromise intrinsic healing capacity.^7^ In addition, the need to preserve intact articular‐sided tendon attachment restricts suture placement and reduces tendon‐bone contact area, potentially limiting the effectiveness of conventional repair strategies.^8^

The technique described in this article addresses these challenges by introducing minced autologous glenohumeral ligament tissue as a biological augment within a modified double‐row suture construct. The glenohumeral ligament shares structural and extracellular matrix characteristics with the rotator cuff tendon, making it a biologically relevant autologous graft source.^9^ Harvesting a limited amount of ligament tissue from selected regions of the MGHL or IGHL can be performed safely without compromising shoulder stability, while simultaneously avoiding the cost and immunologic concerns associated with allogeneic or synthetic graft materials.^10^, ^11^ Processing the ligament into particulate grafts allows formation of a 3‐dimensional scaffold that can be delivered arthroscopically and stabilized within a closed suture‐bridge pocket.^12^

Implantation of ligament particles between the decorticated footprint and the bursal‐sided tear creates a mechanically protected and biologically active interpositional layer.^13^, ^14^ The modified double‐row construct provides compression and containment of the particulate graft, minimizing the risk of graft migration or dead space formation.^15^ Preservation of the deep tendon layer allows the graft to be distributed primarily along the bone bed and superficial tear gap, potentially optimizing the local healing environment without disrupting native tendon attachment.^16^, ^17^

Several technical considerations are essential for the successful application of this technique (Table 2). Adequate preoperative imaging is required to exclude patients with significant anterior instability or insufficient ligament tissue. Intraoperatively, careful selection of harvest sites and avoidance of critical stabilizing structures are mandatory. Graft implantation must be completed before final suture tensioning, and incremental delivery under direct arthroscopic visualization is necessary to ensure even distribution. As with other biologic augmentation strategies, the technique is technically demanding and may require advanced arthroscopic skill and efficient team coordination.

**TABLE 2:** Advantages and Disadvantages for Arthroscopic Rotator Cuff Repair Augmented With Autologous Glenohumeral Ligament Tissue Particulates

**Advantages**	**Disadvantages**
The glenohumeral ligament and rotator cuff tendon are both high‐tension dense connective tissues rich in type I collagen, proteoglycans, glycosaminoglycans, and cell‐adhesion molecules, closely resembling the native tissue microenvironment of the tendon‐bone attachment	Implantation must be completed before final suture‐bridge fixation; the limited working time and space may increase the risk of particle malposition, aggregation, or dead space formation if the technique is not performed meticulously
This technique is indicated for bursal‐sided partial‐thickness rotator cuff tears, allowing preservation of the articular‐sided tendon attachment while enabling implantation of a biological augment within the tear gap to balance structural stability and biological repair	This technique remains exploratory and is primarily supported by histologic rationale and limited preliminary clinical experience, with a lack of long‐term randomized controlled trial data
No additional fixation devices or sutures are required for graft stabilization	Preparation of the particulate graft requires additional operative time and assistance
This approach avoids the additional costs associated with commercially available biologic augmentation materials	
The harvested glenohumeral ligament tissue may otherwise contribute to capsular adhesions, and selective resection of redundant tissue may potentially reduce the risk of postoperative shoulder stiffness while optimizing range of motion	

## DISCLOSURES

The authors (H.L., X.W., H.M., W.S.) declare that they have no known competing financial interests or personal relationships that could have appeared to influence the work reported in this paper.
